# Robustness of Machine Learning Predictions for Determining Whether Deep Inspiration Breath-Hold Is Required in Breast Cancer Radiation Therapy

**DOI:** 10.3390/diagnostics15060668

**Published:** 2025-03-10

**Authors:** Wlla E. Al-Hammad, Masahiro Kuroda, Ghaida Al Jamal, Mamiko Fujikura, Ryo Kamizaki, Kazuhiro Kuroda, Suzuka Yoshida, Yoshihide Nakamura, Masataka Oita, Yoshinori Tanabe, Kohei Sugimoto, Irfan Sugianto, Majd Barham, Nouha Tekiki, Miki Hisatomi, Junichi Asaumi

**Affiliations:** 1 Department of Oral and Maxillofacial Radiology, Graduate School of Medicine, Dentistry and Pharmaceutical Sciences, Okayama University, Okayama 700-8558, Japan; wealhammad@just.edu.jo (W.E.A.-H.);; 2 Department of Oral Medicine and Oral Surgery, Faculty of Dentistry, Jordan University of Science and Technology, Irbid 22110, Jordan; 3 Radiological Technology, Graduate School of Health Sciences, Okayama University, Okayama 700-8558, Japan; 4 Department of Radiology, Matsuyama Red Cross Hospital, Matsuyama 790-8524, Japan; 5 Department of Health and Welfare Science, Graduate School of Health and Welfare Science, Okayama Prefectural University, Okayama 719-1197, Japan; 6 Graduate School of Interdisciplinary Sciences and Engineering in Health Systems, Okayama University, Okayama 770-8558, Japan; 7 Department of Oral Radiology, Faculty of Dentistry, Hasanuddin University, Sulawesi 90245, Indonesia; 8 Department of Dentistry and Dental Surgery, College of Medicine and Health Sciences, An-Najah National University, Nablus 44839, Palestine

**Keywords:** breast cancer, radiation therapy, heart dose, cut-off value, machine learning, robustness, instability, F2 score, deep inspiration breath-hold technique, computed tomography

## Abstract

**Background/Objectives:** Deep inspiration breath-hold (DIBH) is a commonly used technique to reduce the mean heart dose (MHD), which is critical for minimizing late cardiac side effects in breast cancer patients undergoing radiation therapy (RT). Although previous studies have explored the potential of machine learning (ML) to predict which patients might benefit from DIBH, none have rigorously assessed ML model performance across various MHD thresholds and parameter settings. This study aims to evaluate the robustness of ML models in predicting the need for DIBH across different clinical scenarios. **Methods**: Using data from 207 breast cancer patients treated with RT, we developed and tested ML models at three MHD cut-off values (240, 270, and 300 cGy), considering variations in the number of independent variables (three vs. six) and folds in the cross-validation (three, four, and five). Robustness was defined as achieving high F2 scores and low instability in predictive performance. **Results**: Our findings indicate that the decision tree (DT) model demonstrated consistently high robustness at 240 and 270 cGy, while the random forest model performed optimally at 300 cGy. At 240 cGy, a threshold critical to minimize late cardiac risks, the DT model exhibited stable predictive power, reducing the risk of overestimating DIBH necessity. **Conclusions**: These results suggest that the DT model, particularly at lower MHD thresholds, may be the most reliable for clinical applications. By providing a tool for targeted DIBH implementation, this model has the potential to enhance patient-specific treatment planning and improve clinical outcomes in RT.

## 1. Introduction

Radiation therapy (RT) is a vital part of breast cancer treatment [1,2]. Traditional RT methods often expose the heart and lungs to high doses of radiation, which can lead to long-term side effects, especially for those with left-sided breast cancer. These complications can significantly affect patient survival and are often observed within 10 years following treatment [3,4]. Previous studies on patients with breast cancer treated with RT have used the mean heart dose (MHD) as a measure of the radiation exposed to the heart [4,5,6,7]. Therefore, researchers now aim to reduce the MHD to improve overall survival rates.

The MHD cut-off value is usually set based on clinical guidelines and research findings [8]. Radiation oncologists and medical physicists work together to determine safe dose limits for critical organs such as the heart [9]. This is especially crucial in left-sided breast cancer because the heart is so close to the treatment area, increasing the risk of cardiac complications. The deep inspiration breath-hold (DIBH) technique is commonly used to reduce the MHD in patients with left-sided breast cancer [10,11,12]. During DIBH, patients inhale a deep breath and hold it during the delivery of radiation. This inflates the lungs and displaces the heart from the treatment area, thereby lowering the heart’s radiation exposure.

To assess its benefits in RT for breast cancer, DIBH is often compared with free-breathing (FB) techniques without DIBH [13]. This comparison helps ensure that the treatment is as safe and effective as possible, but it can be costly and time-consuming for both patients and RT staff [14]. By using machine learning (ML) approaches, RT staff can analyze patient data to predict who will benefit most from DIBH [15,16]. This targeted approach means that only patients likely to see significant advantages will undergo the additional steps required for DIBH, leading to time and cost savings in both the short and long term.

In our previous publication, we showed that ML models could effectively predict the MHD using a specific cut-off value of 300 cGy [15]. However, in clinical practice, multiple cut-off values are often required to address different patients’ needs and different treatment protocols [8]. In this study, we aim to assess whether these ML models can consistently maintain a stable predictive performance [17] across various clinically relevant cut-off values. Additionally, we evaluate the robustness of the models in [18,19] in terms of their ability to adapt to changes in the modeling process, such as variations in the number of independent variables or adjustments to the number of folds in cross-validation (CV). This evaluation is essential to determine how resilient these models are when exposed to different clinical scenarios and how they may influence the management of radiation therapy patients. To the best of our knowledge, no previously published studies have evaluated the robustness of MHD predictions using ML at different cut-off values.

## 2. Materials and Methods

### 2.1. Study Population

Our study comprised 207 patients diagnosed with left-sided breast cancer who underwent field-in-field (FIF) RT with FB at Okayama University Hospital between 2009 and 2016. These patients were selected from consecutive females with left-sided early-stage breast cancer. Exclusion criteria were simultaneous bilateral breast cancer, treatment with regional nodal irradiation, and treatment using hypo-fractionated irradiation. The patients received treatment at our facility using either the conventional FIF with a one-reference-point technique or an innovative FIF approach employing two reference points (FIF-2RP) [20]. All patients were irradiated for the whole breast with 200 cGy per fraction, with 25 fractions for a total of 5000 cGy, after partial breast resection. Eighty-eight patients were irradiated with an additional 1000–1600 cGy boost on the tumor bed. The heart dose during the 5000 cGy irradiation was the subject of this study [15]. Prior to participation, patients provided written informed consent for RT and the use of their de-identified data for scientific analysis. This investigation adhered to the principles outlined in the Declaration of Helsinki, revised in 2013. Approval for utilizing de-identified post-radiation data was obtained from the Ethical Review Board of our institution (approval no. 2103-024).

### 2.2. Data Collection

In March 2021, we retrospectively collected patient data from the RT planning system following computed tomography (CT) simulations. Key parameters, including breast separation (SEP), chest wall thickness (CWT), and the MHD, were carefully documented. SEP and CWT were evaluated for each patient using single-slice CT images taken at the nipple level as shown in Figure S1. SEP was defined as the distance along the posterior edge of the tangent fields, while CWT represents the distance from the nipple surface to the lung, measured perpendicularly to SEP, as described in a previous study [15]. Additionally, we retrieved demographic and clinical information from each patient’s medical records, including their age and body mass index (BMI), the tumor location, and the specific RT technique employed. Table 1 summarizes the patient characteristics.

### 2.3. ML Models

In this study, we utilized Anaconda Python version 3.9, along with various Python libraries (Python Software Foundation, Wilmington, DE, USA), to develop and experiment with our ML models. A total of ten supervised ML models were employed to accurately classify patients into low- or high-MHD categories based on predefined cut-off values. The models included gradient boosting (GB), decision tree (DT), bagging, deep neural network (DNN), random forest (RF), K-nearest neighbor (KNN), support vector machine (SVM), naïve Bayes (NB), logistic regression (LR), and ridge classifier (RC) models. These models were used to identify relationships and dependencies between the dependent variable (MHD) and the independent variables (SEP, CWT, age, BMI, tumor location, and RT method), enabling the prediction of a high or low MHD based on patterns learned from the training dataset.

Additionally, to address the class imbalance in the training data, we applied the synthetic minority over-sampling technique (SMOTE) in conjunction with the “imblearn” pipeline to increase the representation of underrepresented high- or low-MHD patients [21].

### 2.4. Model-Building Process

The model-building process involved exploring various configurations encompassing changes in the number of independent variables, classification cut-off values, and the number of folds used in the grid-search CV (GridSearchCV) process. Two primary configurations were considered: one utilizing three independent variables (SEP, CWT, and BMI) and the other incorporating six independent variables (SEP, CWT, BMI, age, tumor location, and RT method). Furthermore, the classification cut-off values of 240, 270, and 300 cGy were evaluated, alongside the number of folds in GridSearchCV (three, four, and five). The dataset at 240 cGy is summarized in File S1.

This comprehensive approach resulted in the creation of eighteen distinct sub-models for each ML model, each tailored to a specific setting.

**Figure 1:**
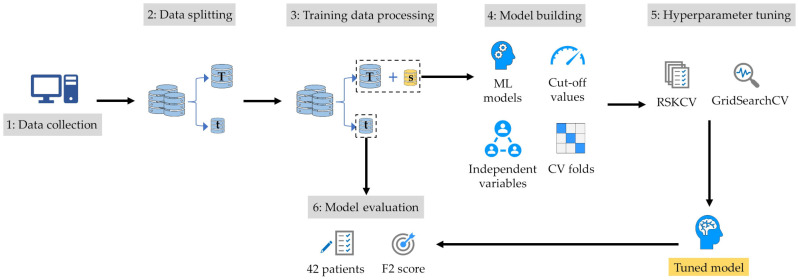
Overview of criteria of building models. T: training dataset; t: test dataset; s: synthetic minority over-sampling set; ML: machine learning; CV: cross-validation; RSKCV: repeated stratified K-fold cross-validation; GridSearchCV: grid-search cross-validation.

A general overview of the model building process is provided in Figure 1. The first step involved randomly splitting the dataset into training and test sets in an 80:20 ratio. Due to the imbalanced nature of the dataset, a stratified split was used to ensure that the proportion of patients in each class (low and high MHD) was consistent across both the original dataset and the partitions. This led to an 80% representation of each class in the training set and 20% in the test set [22]. This approach was selected for its ability to preserve data integrity while effectively managing class imbalances within the dataset.

The next step involved fine-tuning the parameters of each model using the training dataset through a hyperparameter tuning process. With the primary goal of accurately identifying patients who might not require DIBH, our focus was on minimizing false negatives (i.e., patients incorrectly classified as having a low MHD). To achieve this, the models were trained using the F2 score as the primary performance metric within a GridSearchCV framework, as the F2 score places a greater emphasis on minimizing false negatives. In the next step, the models were built using the optimal hyperparameters determined from this tuning process.

In our study, hyperparameter tuning was conducted using repeated stratified K-fold CV (RSKCV), a technique employed to enhance the reliability of model performance evaluation [23]. RSKCV involves systematically partitioning the dataset into K folds while maintaining a consistent distribution of classes in each fold. This process is repeated multiple times to mitigate variability in performance estimates. Specifically, we utilized RSKCV within the GridSearchCV framework to evaluate various hyperparameter configurations. By repeatedly sampling and stratifying the data, RSKCV ensured a robust assessment of model performance, aiding in the selection of hyperparameters that generalize effectively to unseen data. This approach was pivotal in optimizing our models’ performance while minimizing the risk of overfitting [24].

Notably, to address potential biases introduced by synthetic high- or low-MHD patients generated through SMOTE, these synthetic instances were exclusively added into the training folds—not into the validation folds—using an “imblearn” pipeline. This measure ensured that the validation of our models relied solely on real data.

The code for hyperparameter tuning is shown in File S2.

### 2.5. Model Evaluation

To rigorously assess the models’ performance, a comprehensive external evaluation was conducted using an independent test set comprising 42 patients who were entirely distinct from those involved in model training and construction. This external validation step ensured that the models’ effectiveness transcended the confines of the training data and accurately reflected their real-world utility.

During this evaluation, the classification cut-off value was systematically varied to encompass clinically relevant thresholds: MHD ≥ 240 cGy; MHD ≥ 270 cGy; and MHD ≥ 300 cGy. This approach allowed for a nuanced analysis of the models’ performance across different levels of sensitivity and specificity, catering to diverse clinical needs and scenarios.

The primary metric used for assessing each model’s performance was the F2 score [15], chosen for its ability to strike a balanced evaluation between precision and recall, with a specific focus on minimizing false negatives, a critical consideration in medical decision making. Significant differences in the F2 scores among the models were analyzed with the permutation test using R version 4.3.2 (R Core Team) and the “stats” package. Values of *p* < 0.05 were considered statistically significant. Model instability, defined as the difference between the minimum and maximum F2 scores, was assessed using the median instability value for each cut-off value. Models were categorized as having “high” or “low” instability if their instability exceeded or fell below the median value, respectively, for each cut-off value.

By leveraging this robust evaluation framework, we aimed to provide comprehensive insights into the models’ efficacy and generalizability, thereby bolstering confidence in their real-world deployment and clinical impact.

The code for the best performance results is shown in File S3.

### 2.6. Predicted DIBH

To accurately assess the differences between the predicted and real incidences of DIBH at different radiation doses, we conducted a comparative analysis. This involved creating a graph that showed both the predicted and actual percentages of patients needing DIBH in the test set.

For this analysis, we followed these steps:

We selected the best-performing model at each classification cut-off value.

Using Formula (1), we recorded the actual percentages of patients requiring DIBH (real DIBH) for the best-performing model:

Real DIBH = ((TP + FN)/(total patients in the test set)) × 100%(1)

where TP represents true positives, and FN represents false negatives.

Using Formula (2), we calculated the predicted percentages of patients needing DIBH (predicted DIBH) for the best-performing model.

Predicted DIBH = ((TP + FP)/(total patients in the test set)) × 100%(2)

where FP represents false positives.

Finally, we plotted the real and predicted percentages of patients needing DIBH for each classification cut-off value to visualize and compare the discrepancies.

## 3. Results

### 3.1. Patient Characteristics

The characteristics of patients who were involved in this study are shown in Table 1.

### 3.2. Model Performance and Robustness

In this study, we created models by adjusting different factors such as the classification cut-off value, the number of independent variables, and the folds in CV. Table 2 shows the F2 scores and the predictive performance of the models under these different conditions. Additionally, Table 3 presents the results of pairwise permutation tests between models using different cut-off values. The numbers in Table 3 indicate the *p*-values of pairwise permutation tests. The robustness of the ML models was evaluated across different cut-off values (240, 270, and 300 cGy) based on the models’ median F2 scores and instability metrics.

The median instability value was 0.100, 0.121, and 0.255 for cut-off values of 240, 270, and 300 cGy, respectively.

At a cut-off value of 240 cGy, GB demonstrated superior performance, with the highest median F2 score of 0.846, but also exhibited the highest model instability of 0.454. DT showed consistent performance, with the second-highest median F2 score of 0.701, and low instability (0.038). Bagging had the third-highest median F2 score (0.683) but with high instability (0.174). Based on Table 3, no significant difference in the median F2 score was observed among GB, DT, and bagging. As a result, DT was the most robust model at the cut-off value of 240 cGy.

At 270 cGy, DT achieved the highest median F2 score, 0.795, and showed notable robustness, achieving the lowest model instability (0.018). GB achieved the second-highest median F2 score, 0.735, but the highest instability (0.823). Therefore, DT was the most robust model at the cut-off value of 270 cGy.

For the cut-off value of 300 cGy, bagging and KNN exhibited the highest (0.789) and third-highest (0.750) median F2 scores but with high instability. In contrast, RF showed the second-highest median F2 score of 0.756, with low instability (0.089). No significant differences in the median F2 score were observed among bagging, KNN, and RF. As a result, RF was the most robust model at the cut-off value of 300 cGy.

### 3.3. Comparison Between Predicted DIBH and Real DIBH

**Figure 2:**
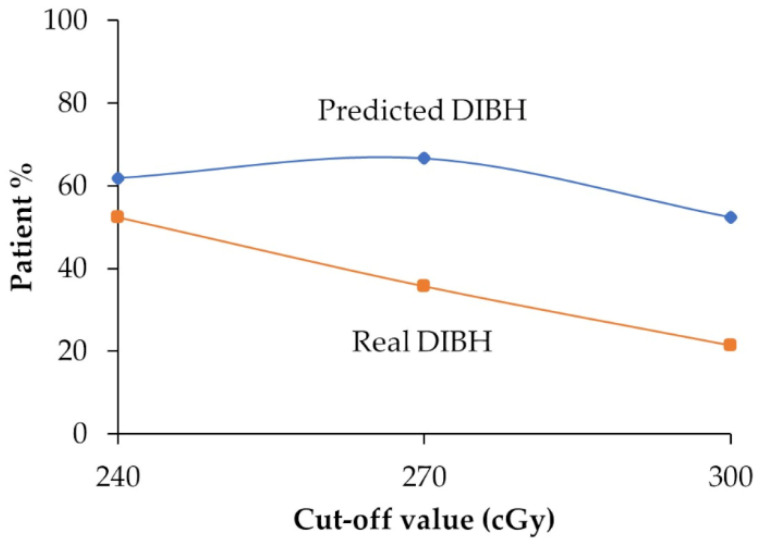
The discrepancies between the predicted and real incidences of deep inspiration breath-hold across different radiation doses. DIBH: deep inspiration breath-hold.

Figure 2 presents a comparison between the predicted and real percentages of patients requiring DIBH using the best-performing model at each classification cut-off value: DT at 240 and 270 cGy and RF at 300 cGy. This analysis reveals the discrepancies between the predicted and actual incidences of DIBH across different radiation doses.

The graph indicates that the models tended to overestimate DIBH incidences compared to actual patient data across all cut-off values. However, at 240 cGy, the model showed only a subtle discrepancy of 9.5% between the predicted and actual DIBH incidences, while it demonstrated a 31.0% discrepancy at 270 cGy and 31.0% at 300 cGy.

## 4. Discussion

In this study, we evaluated the robustness and stability of ML models for identifying patients who may not require DIBH across various classification cut-off values. Additionally, we examined the effect of altering the number of independent variables and the number of CV folds on model performance. We identified the most robust ML models as DT (at cut-off values of 240 and 270 cGy) and RF (at 300 cGy) based on their high median F2 scores and low instability. In contrast, GB was not considered robust due to its high instability, despite achieving a high median F2 score at 240 and 270 cGy.

The choice of ML models for predicting the need for DIBH in breast cancer RT is based on their ability to handle complex and multidimensional data. KNN and the DNN are particularly effective for modeling nonlinear relationships, such as those between the BMI and SEP in influencing heart dose [15], while LR is strong for binary classification tasks [25]. RF enhances stability and accuracy by leveraging an ensemble approach, improving predictions in complex datasets [26]. NB is well-suited for small datasets, offering reliable performance despite limited data availability [27].

The reliability and robustness of ML models are critical considerations in clinical settings [28]. These models are typically trained and optimized under specific conditions, including fixed parameter settings and consistent data distributions. However, their performance may vary when applied to new conditions or when key parameters are adjusted [29]. Variations in patient demographics, data quality, or disease prevalence can all influence model accuracy and reliability [30]. Bouthillier et al. highlighted challenges related to reproducibility in ML research, emphasizing the importance of standardized evaluation protocols to ensure robustness across diverse conditions [31]. Additionally, Goodfellow et al. demonstrated how slight changes in input data can significantly affect model predictions, underscoring the need for robust training methods [32].

To comprehensively assess model robustness, we evaluated model instability by calculating the range of F2 scores across six sub-models constructed for each cut-off value [17]. This measure underscores the sensitivity of ML models to parameter changes and emphasizes the necessity of rigorous evaluation to ensure consistent clinical performance.

In clinical practice, having stable and reliable ML models across different cut-off values is paramount [33]. Cut-off values often determine critical decision thresholds such as treatment recommendations. Instability in model performance at different cut-off values can lead to inconsistent clinical decisions, potentially compromising patient safety and treatment outcomes. Kamizaki et al. identified the DNN as the optimal algorithm for DIBH prediction, achieving an F2 score of 0.80 [34], while KNN was the best-performing model in one of our studies, with an F2 score of 0.67 [15]. However, our study emphasizes that model robustness under varying constraints is more clinically relevant than merely achieving the highest performance metrics. While the DNN in Kamizaki’s study demonstrated superior performance, our findings highlight the importance of stability, ensuring that consistent and dependable decision making is possible even under different clinical conditions. Healthcare professionals can trust predictions and recommendations made by ML systems using reliable models, regardless of minor variations in input parameters. By developing and validating models that demonstrate robustness across a range of cut-off values, we can enhance the dependability of ML applications in RT, ultimately improving patient management and health outcomes.

In our study, DT at 240 and 270 cGy and RF at 300 cGy emerged as the most robust ML models, consistently achieving high median F2 scores and low instability. This aligns with the literature, which highlights the robustness and consistency of DT [35] and RF [26] models in predictive modeling applications. In contrast, at 240 and 270 cGy, the GB model exhibited the highest instability among all models. This instability can be attributed to several factors inherent to GB algorithms. GB might be prone to overfitting, particularly in small or noisy datasets, which can result in fluctuations in performance when subjected to various changes in data or parameter settings [36]. The sequential nature of GB, which builds an ensemble of weak learners to correct errors incrementally, further contributes to its sensitivity to data variations [37].

In this research, we selected three cut-off values of 240, 270, and 300 cGy. Following the International Quantitative Analysis of Normal Tissue Effects in the Clinic (QUANTEC) guidelines [7], using a high cut-off value such as 300 cGy, as previously reported [15], would result in fewer patients being treated with DIBH compared with using lower cut-off values such as 240 or 270 cGy. Consequently, the number of late cardiac side effects might increase for patients treated with the higher cut-off value, although these values should be selected based on several guidelines for breast cancer RT. Our evaluation of model performance revealed a discrepancy between real and predicted DIBH outcomes across three cut-off values. This sensitivity underscores the critical impact of threshold selection on predictive accuracy. Throughout our analysis, the model consistently tended to overestimate the necessity of DIBH, potentially resulting in misclassifications. However, we found the 240 cGy cut-off value to be particularly promising for DIBH predictions. DT, our best-performing model at this cut-off value, achieved a high median F2 score (0.701) with low model instability (0.038). In particular, DT exhibited a minimal discrepancy of only 9.5% between real and predicted DIBH incidences at this cut-off value. These results underscore the 240 cGy threshold as the most accurate and suitable for clinical application.

A major limitation of our study is its retrospective design and the specific group of patients that we included. Our dataset might not represent all breast cancer patients because it originates from a single hospital and may have selection biases. Additionally, we used specific techniques (FIF-2RP) that might not be used in other hospitals. Another limitation is the small, imbalanced dataset, which may constrain the robustness and generalizability of our models. To minimize biased performance estimates, we applied RSKCV exclusively to the training set and evaluated our models on a small, independent test set of unseen data. Nevertheless, the computational constraints of our study limited us from employing bootstrapping with optimism correction, which could have provided a more robust assessment of model performance. While, in our study, we considered the MHD as the primary factor influencing the decision to use DIBH, clinical practice also considers omics features like HER2 expression, which can influence decisions on the use of Herceptin, which has associated cardiac risks. A notable study, the CHECK HEART-BC study, found that 8.5% of breast cancer patients developed cardiomyopathy, with the concurrent use of trastuzumab and radiotherapy identified as significant risk factors contributing to this adverse outcome [38]. Therefore, other omics features beyond the MHD may also affect the decision to use DIBH in practice. Our study focused on variables available from single-slice CT scans for their convenience in daily clinical practice, including CWT and SEP. However, incorporating additional volumetric variables from multi-slice CT, such as heart volume in the field, lung volume changes, and maximum heart depth [39], might potentially enhance model accuracy. More studies with larger, multi-institutional groups and a prospective design are needed to confirm our models’ reliability and clinical usefulness.

## 5. Conclusions

In summary, our study shows the importance of evaluating the robustness and reliability of ML models in predicting the need for DIBH in patients receiving RT for left-sided breast cancer. We found that DT and RF emerged as the top-performing models in our study, demonstrating a consistent and reliable performance across various conditions. Despite the limitations of our retrospective, single-institution study, our findings provide useful insights for improving ML models for clinical decision making.

## Figures and Tables

**Table 1:** Patient characteristics.

Characteristic	Value
Age (mean ± SD, years)	55.3 ± 11.1
Body mass index (mean ± SD)	22.9 ± 3.9
Tumor location (%)	
Upper-inner quadrant	27.1%
Lower-inner quadrant	9.2%
Upper-outer quadrant	51.7%
Lower-outer quadrant	4.8%
Central portion	7.2%
Radiation method (%)	
FIF-1RP	33.8%
FIF-2RP	66.2%
Breast separation (mean ± SD, cm)	18.8 ± 2.6
Chest wall thickness (mean ± SD, cm)	6.0 ± 1.2
Mean heart dose (mean ± SD, cGy)	251 ± 81
High	106 ^*1^, 74 ^*2^, 43 ^*3^
Low	101 ^*1^, 133 ^*2^, 164 ^*3^

SD: standard deviation. The body mass index was calculated as weight (kg)/height^2^ (m). Tumor location was determined according to the International Classification of Diseases for Oncology, Third Edition (ICD-O-3). The radiation method (n) refers to the number of patients treated using either the field-in-field technique with one reference point or two reference points. Breast separation (cm) was measured as the distance along the posterior edge of the tangent fields at the nipple level, while chest wall thickness (cm) was defined as the distance from the skin surface to the lung, also measured at the nipple level. A high mean heart dose was defined as a value equal to or greater than the designated cut-off, whereas a low mean heart dose was classified as any value below this threshold. ^*1^: the number of patients at the 240 cGy cut-off value. ^*2^: the number of patients at the 270 cGy cut-off value. ^*3^: the number of patients at the 300 cGy cut-off value.

**Table 2:** F2 scores and predictive performance of models.

Cut-Off Value	# of Variables	Folds	GB	DT	Bagging	DNN	RF	KNN	SVM	NB	LR	RC
240 cGy	3 variables	3-fold	0.846	0.701	0.707	0.601	0.607	0.528	0.560	0.528	0.528	0.485
		4-fold	0.846	0.701	0.707	0.560	0.663	0.528	0.560	0.528	0.528	0.485
		5-fold	0.392	0.701	0.714	0.636	0.607	0.607	0.560	0.528	0.485	0.485
	6 variables	3-fold	0.846	0.739	0.582	0.652	0.566	0.571	0.681	0.550	0.544	0.544
		4-fold	0.799	0.701	0.660	0.619	0.571	0.630	0.544	0.594	0.588	0.544
		5-fold	0.846	0.701	0.540	0.625	0.648	0.630	0.544	0.594	0.544	0.544
	Median		0.846	0.701	0.683	0.622	0.607	0.589	0.560	0.539	0.536	0.514
	Q1		0.811	0.701	0.602	0.606	0.580	0.539	0.548	0.528	0.528	0.485
	Q3		0.846	0.701	0.707	0.633	0.638	0.624	0.560	0.583	0.544	0.544
	IQR		0.035	0.000	0.106	0.028	0.058	0.085	0.012	0.055	0.016	0.059
	Maximum		0.846	0.739	0.714	0.652	0.663	0.630	0.681	0.594	0.588	0.544
	Minimum		0.392	0.701	0.540	0.560	0.566	0.528	0.544	0.528	0.485	0.485
	Model instability		0.454 *	0.038	0.174 *	0.092	0.097	0.102 *	0.137 *	0.066	0.103 *	0.059
270 cGy	3 variables	3-fold	0.735	0.786	0.687	0.625	0.731	0.679	0.555	0.679	0.679	0.679
		4-fold	0.735	0.786	0.687	0.632	0.823	0.679	0.722	0.679	0.731	0.679
		5-fold	0.823	0.786	0.679	0.625	0.687	0.670	0.740	0.679	0.679	0.679
	6 variables	3-fold	0.735	0.804	0.625	0.523	0.687	0.705	0.714	0.639	0.609	0.555
		4-fold	0.000	0.804	0.740	0.641	0.687	0.654	0.632	0.639	0.609	0.555
		5-fold	0.804	0.804	0.687	0.555	0.687	0.639	0.647	0.639	0.555	0.555
	Median		0.735	0.795	0.687	0.625	0.687	0.674	0.680	0.659	0.644	0.617
	Q1		0.735	0.786	0.681	0.573	0.687	0.658	0.636	0.639	0.609	0.555
	Q3		0.787	0.804	0.687	0.630	0.720	0.679	0.720	0.679	0.679	0.679
	IQR		0.052	0.018	0.006	0.058	0.033	0.021	0.084	0.040	0.070	0.124
	Maximum		0.823	0.804	0.740	0.641	0.823	0.705	0.740	0.679	0.731	0.679
	Minimum		0.000	0.786	0.625	0.523	0.687	0.639	0.555	0.639	0.555	0.555
	Model instability		0.823 *	0.018	0.115	0.118	0.136 *	0.066	0.185 *	0.040	0.176 *	0.124 *
300 cGy	3 variables	3-fold	0.603	0.725	0.789	0.689	0.737	0.762	0.762	0.714	0.714	0.714
		4-fold	0.762	0.725	0.789	0.789	0.775	0.775	0.762	0.714	0.714	0.714
		5-fold	0.576	0.306	0.510	0.689	0.775	0.775	0.750	0.714	0.701	0.714
	6 variables	3-fold	0.727	0.666	0.714	0.526	0.775	0.737	0.409	0.535	0.526	0.526
		4-fold	0.520	0.689	0.803	0.454	0.686	0.737	0.545	0.535	0.526	0.526
		5-fold	0.510	0.666	0.803	0.614	0.737	0.517	0.526	0.614	0.526	0.526
	Median		0.590	0.678	0.789	0.652	0.756	0.750	0.648	0.664	0.614	0.620
	Q1		0.534	0.666	0.733	0.548	0.737	0.737	0.531	0.555	0.526	0.526
	Q3		0.696	0.716	0.800	0.689	0.775	0.772	0.759	0.714	0.711	0.714
	IQR		0.162	0.050	0.067	0.141	0.038	0.035	0.228	0.159	0.185	0.188
	Maximum		0.762	0.725	0.803	0.789	0.775	0.775	0.762	0.714	0.714	0.714
	Minimum		0.510	0.306	0.510	0.454	0.686	0.517	0.409	0.535	0.526	0.526
	Model instability		0.252	0.419 *	0.293 *	0.335 *	0.089	0.258 *	0.353 *	0.179	0.188	0.188

# of variables: number of variables. GB: gradient boosting; DT: decision tree; DNN: deep neural network; RF: random forest; KNN: K-nearest neighbor; SVM: support vector machine; NB: naïve Bayes; LR: logistic regression; RC: ridge classifier. Q1: first quartile, represents the 25th percentile; Q3: third quartile, represents the 75th percentile; IQR: interquartile range, calculated as Q3–Q1. Model instability is the variation in model F2 scores under different conditions, calculated as the difference between the maximum and minimum values for each model. * indicates a “significantly high” instability value, surpassing the median instability values of the models for each respective cut-off. Blue values are the highest median F2 scores at each cut-off value. Red values are the highest instability values associated with the highest median F2 scores.

**Table 3:** Pairwise permutation tests between models with various cut-off values.

Cut-off value = 240 cGy										
Model	GB	DT	Bagging	DNN	RF	KNN	SVM	NB	LR	RC
GB	N/A									
DT	0.61	N/A								
Bagging	0.206	0.121	N/A							
DNN	0.102	0.002 *	0.292	N/A						
RF	0.08	0.002 *	0.26	0.807	N/A					
KNN	0.035 *	0.002 *	0.087	0.186	0.294	N/A				
SVM	0.052	0.002 *	0.061	0.132	0.225	0.82	N/A			
NB	0.015 *	0.002 *	0.032 *	0.013 *	0.026 *	0.253	0.539	N/A		
LR	0.015 *	0.002 *	0.015 *	0.004 *	0.006 *	0.082	0.143	0.409	N/A	
RC	0.015 *	0.002 *	0.009 *	0.002 *	0.002 *	0.024 *	0.022 *	0.069	0.364	N/A
Cut-off value = 270 cGy										
Model	GB	DT	Bagging	DNN	RF	KNN	SVM	NB	LR	RC
GB	N/A									
DT	0.067	N/A								
Bagging	0.994	0.002 *	N/A							
DNN	0.905	0.002 *	0.011 *	N/A						
RF	0.944	0.015 *	0.349	0.002 *	N/A					
KNN	1	0.002 *	0.496	0.004 *	0.058	N/A				
SVM	0.994	0.002 *	0.66	0.058	0.217	0.937	N/A			
NB	1	0.002 *	0.197	0.009 *	0.002 *	0.372	0.76	N/A		
LR	1	0.002 *	0.195	0.223	0.048 *	0.351	0.55	0.63	N/A	
RC	0.955	0.002 *	0.054	0.649	0.002 *	0.139	0.225	0.182	0.589	N/A
Cut-off value = 300 cGy										
Model	GB	DT	Bagging	DNN	RF	KNN	SVM	NB	LR	RC
GB	N/A									
DT	0.887	N/A								
Bagging	0.097	0.251	N/A							
DNN	0.883	0.985	0.132	N/A						
RF	0.024 *	0.013 *	0.924	0.048 *	N/A					
KNN	0.132	0.364	0.619	0.182	0.727	N/A				
SVM	0.887	0.974	0.175	1	0.128	0.336	N/A			
NB	0.714	0.924	0.147	0.82	0.022 *	0.149	0.903	N/A		
LR	0.981	0.972	0.093	0.935	0.013 *	0.069	0.97	0.589	N/A	
RC	0.948	0.981	0.095	0.952	0.022 *	0.069	0.987	0.61	1	N/A
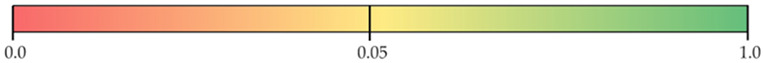										

GB: gradient boosting; DT: decision tree; DNN: deep neural network; RF: random forest; KNN: K-nearest neighbor; SVM: support vector machine; NB: naïve Bayes; LR: logistic regression; RC: ridge classifier. N/A: not applicable. The numbers indicate the *p*-values of pairwise permutation tests. * indicates *p* < 0.05 in permutation tests between each pairwise model.

## Data Availability

The original contributions presented in this study are included in the article/Supplementary Material. Further inquiries can be directed to the corresponding author.

## Supplementary Materials

The following supporting information can be downloaded at https://www.mdpi.com/article/10.3390/diagnostics15060668/s1. Figure S1: Single-slice CT parameters; File S1: Dataset at 240 cGy; File S2: Hyperparameter tuning (DT); File S3: Performance results (DT).
