# Supplementary figures and images for: Robustness of Machine Learning Predictions for Determining Whether Deep Inspiration Breath-Hold Is Required in Breast Cancer Radiation Therapy
Source: Diagnostics (Basel). 2025 Mar 10;15(6):668. doi: 10.3390/diagnostics15060668 (PMC11941375; doi:10.3390/diagnostics15060668)

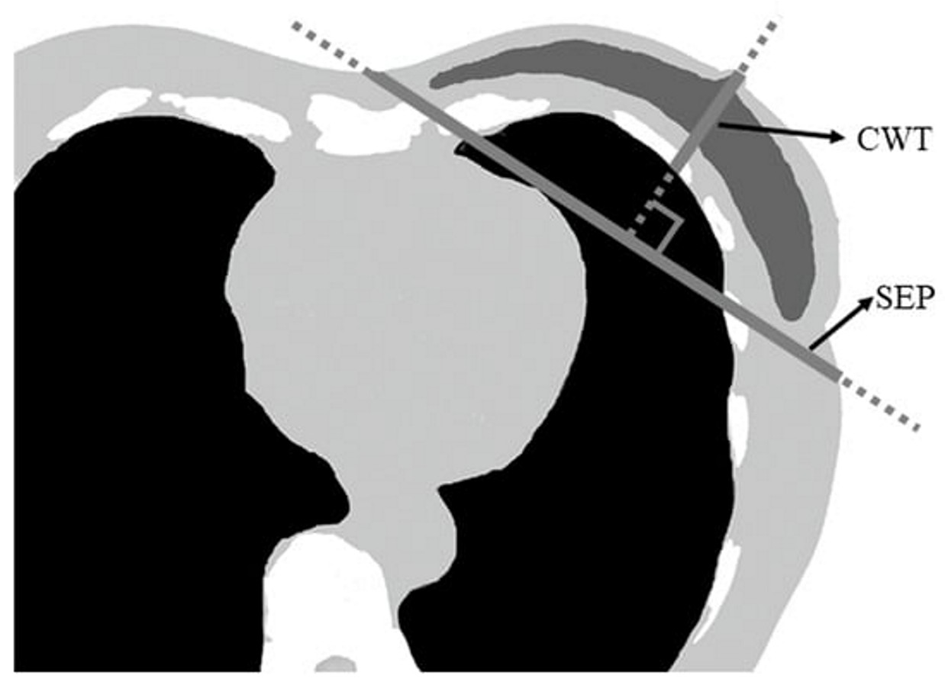

Supplement: Supplementary file 1 [file diagnostics-15-00668-s001.zip › Figure S1.TIFF]
